# A bibliometric analysis of research on cognitive function and carotid atherosclerosis: global trends and hotspots

**DOI:** 10.3389/fneur.2025.1644172

**Published:** 2026-01-13

**Authors:** Qi Sun, Fanfan Zheng, Guopeng Chen, Liuyin Jin

**Affiliations:** 1 The Affiliated Lihuili Hospital of Ningbo University, Ningbo, Zhejiang, China; 2 Hubei Provincial Hospital of Integrated Chinese & Western Medicine, Wuhan, Hubei, China; 3 Zhujiang Hospital of Southern Medical University, Guangzhou, Guangdong, China; 4 Guangdong-Hong Kong-Macao Greater Bay Area Center for Brain Science and Brain-Inspired Intelligence, Southern Medical University, Guangzhou, Guangdong, China; 5 The Second People's Hospital of Lishui, Lishui, Zhejiang, China

**Keywords:** cognitive function, carotid atherosclerosis, bibliometrics, neuroinflammation, visual analysis

## Abstract

**Background:**

Cognitive impairment associated with carotid atherosclerosis has attracted growing attention in recent years due to its implications in neurodegenerative diseases and vascular dementia. However, the knowledge structure and research trends in this interdisciplinary field remain unclear.

**Methods:**

This study conducted a comprehensive bibliometric analysis based on the Web of Science Core Collection and Scopus databases. Publications from 1995 to 2025 related to “cognitive function” and “carotid atherosclerosis” were retrieved, yielding 3,823 unique articles after deduplication using the bibliometrix R package. CiteSpace, VOSviewer, and ggplot2 were applied to visualize publication trends, co-authorship, co-citation networks, keyword clustering, and burst detection.

**Results:**

The number of publications has increased steadily since 2005, with a notable acceleration after 2016. China and the United States are the leading contributors, with Capital Medical University and Harvard Medical School as prominent institutions. Highly cited papers are primarily guideline-based or cohort studies. Keyword clustering revealed three major thematic areas: (1) clinical assessment and epidemiological features of cognitive impairment, (2) neurodegenerative and vascular dementia mechanisms, and (3) cellular injury pathways involving inflammation and oxidative stress. Recent citation bursts of keywords such as “microglia,” “postoperative cognitive dysfunction,” and “cerebral small vessel disease” indicate a shift toward translational and clinical research. Animal models (e.g., bilateral carotid artery occlusion) and mechanistic studies on neuroinflammation, blood–brain barrier dysfunction, and microglial activation have emerged as key areas of focus.

**Conclusion:**

This bibliometric study outlines the evolution, hotspots, and future directions of research on cognitive function and carotid atherosclerosis. While research output has grown significantly, particularly in China, international collaboration remains uneven. Emerging evidence highlights the critical role of vascular pathology and neuroinflammation in cognitive decline, offering new avenues for diagnosis and intervention. Limitations include potential database bias and a lack of qualitative content assessment. Future work should integrate broader data sources and in-depth analyses to enhance the understanding of this complex domain.

## 1 Introduction

Cognitive function refers to the brain’s ability to process and interpret information, encompassing aspects such as sensory-perceptual processing, language learning and memory, cognitive flexibility, executive functioning, general intelligence, processing speed, and working memory. It serves as the foundation for individuals to engage in daily learning, work, and social interactions (1). Normal cognitive function enables individuals to understand their environment, formulate plans, solve problems, and adapt to changes. Cognitive health is a crucial factor in maintaining quality of life and independence (2). With advancing age, cognitive capacity tends to decline progressively (3). In recent years, the global aging trend has intensified, leading to a steady increase in the prevalence of cognitive impairment. According to the World Health Organization, approximately 50 million people currently suffer from some form of cognitive impairment worldwide, and this number is projected to rise to 152 million by 2050 (4). Cognitive impairment often manifests as memory decline, decreased attention, impaired language and executive abilities, and in severe cases may progress to dementia, substantially affecting quality of life and social participation (5). Growing evidence suggests that cardiovascular and cerebrovascular factors play a key role in the development of cognitive dysfunction (6, 7). Among them, carotid atherosclerosis- a typical manifestation of atherosclerosis in the carotid artery system- has drawn increasing attention. It is characterized by arterial wall thickening, plaque formation, and restricted blood flow, which can compromise cerebral perfusion and contribute to cognitive decline. Numerous studies have demonstrated a strong association between carotid pathology and deteriorating cognitive performance (8–10). Additionally, cognitive improvements have been reported following carotid stenting or endarterectomy procedures (11–14). Clinically, carotid atherosclerosis is commonly assessed via carotid intima-media thickness (IMT), while plaque burden is also considered a significant predictor of cerebral small vessel disease and cognitive impairment. Rayan Anbar et al. investigated the association between carotid atherosclerosis and cognitive decline and found that, although limited cross-sectional evidence links IMT to lower cognitive performance as assessed by the Mini-Mental State Examination, the evaluation of plaque-related cognitive impact remains imprecise (15). Despite the increasing number of studies in this area, the literature remains fragmented, and a systematic and quantitative synthesis is still lacking.

Bibliometrics, as a methodology grounded in statistics and information science, provides a systematic approach for analyzing and quantifying large bodies of literature. This method is widely used to uncover developmental trajectories, research hotspots, collaboration networks, and emerging trends within specific scientific domains. Through keyword co-occurrence analysis, co-citation analysis, and the construction of institutional and author collaboration networks, bibliometrics offers researchers a comprehensive and objective academic perspective (16, 17). Although increasing attention has been directed toward the intersection between cognitive function and carotid atherosclerosis in recent years, a systematic bibliometric analysis dedicated to this emerging field is still lacking. Existing studies predominantly focus on related areas such as vascular cognitive impairment. Although increasing attention has been directed toward the intersection between cognitive function and carotid atherosclerosis in recent years, a systematic bibliometric analysis dedicated to this emerging field is still lacking. Existing studies predominantly focus on related areas such as vascular cognitive impairment (18), dementia, and cerebral microbleeds or stroke (17, 19, 20), but have not approached the topic from a disease-specific perspective centered on “cognitive impairment caused by carotid atherosclerosis.” These earlier works fail to systematically map the knowledge structure, thematic evolution, or translational trends of this specific intersection. In addition, the most influential literature to date consists largely of clinical guidelines for hypertension, stroke, and atherosclerotic disease, or broader studies on cardiovascular and neurodegenerative conditions. While these publications provide valuable background for understanding the relationship between vascular pathology and cognitive decline, they do not offer a bibliometric delineation of the overall landscape of the “carotid atherosclerosis × cognitive function” research domain. Consequently, the field still lacks a clear and structured understanding of its knowledge network, developmental trajectory, and global research collaboration patterns. To address this gap, the present study is the first to treat “cognitive impairment related to carotid atherosclerosis” as an independent research theme. We integrated English-language publications indexed in the Web of Science Core Collection and Scopus from 1995 to 2025 and applied multiple bibliometric tools, including CiteSpace and VOSviewer, to analyze global publication trends, keyword evolution, collaboration networks, core authors, and highly cited literature. The novelty of this work lies in three main aspects: (1) it represents the first comprehensive bibliometric investigation of the “carotid atherosclerosis × cognitive function” field from a disease-specific perspective, rather than embedding it within broader categories such as vascular cognitive impairment or neurodegenerative disorders; (2) it systematically delineates the thematic evolution of the field, spanning pathological mechanisms, vascular risk factors, neuroimaging assessment, and neurovascular unit–related pathways; (3) it highlights an emerging interdisciplinary trend in which research progresses from basic mechanistic exploration toward clinical interventions and precision medicine. Overall, this study aims to construct a holistic knowledge map of the field, clarify research hotspots and methodological gaps, and provide a conceptual framework to guide future multidisciplinary efforts from mechanistic studies to clinical translation.

## 2 Methods

### 2.1 Data source and search strategy

**Figure 1:**
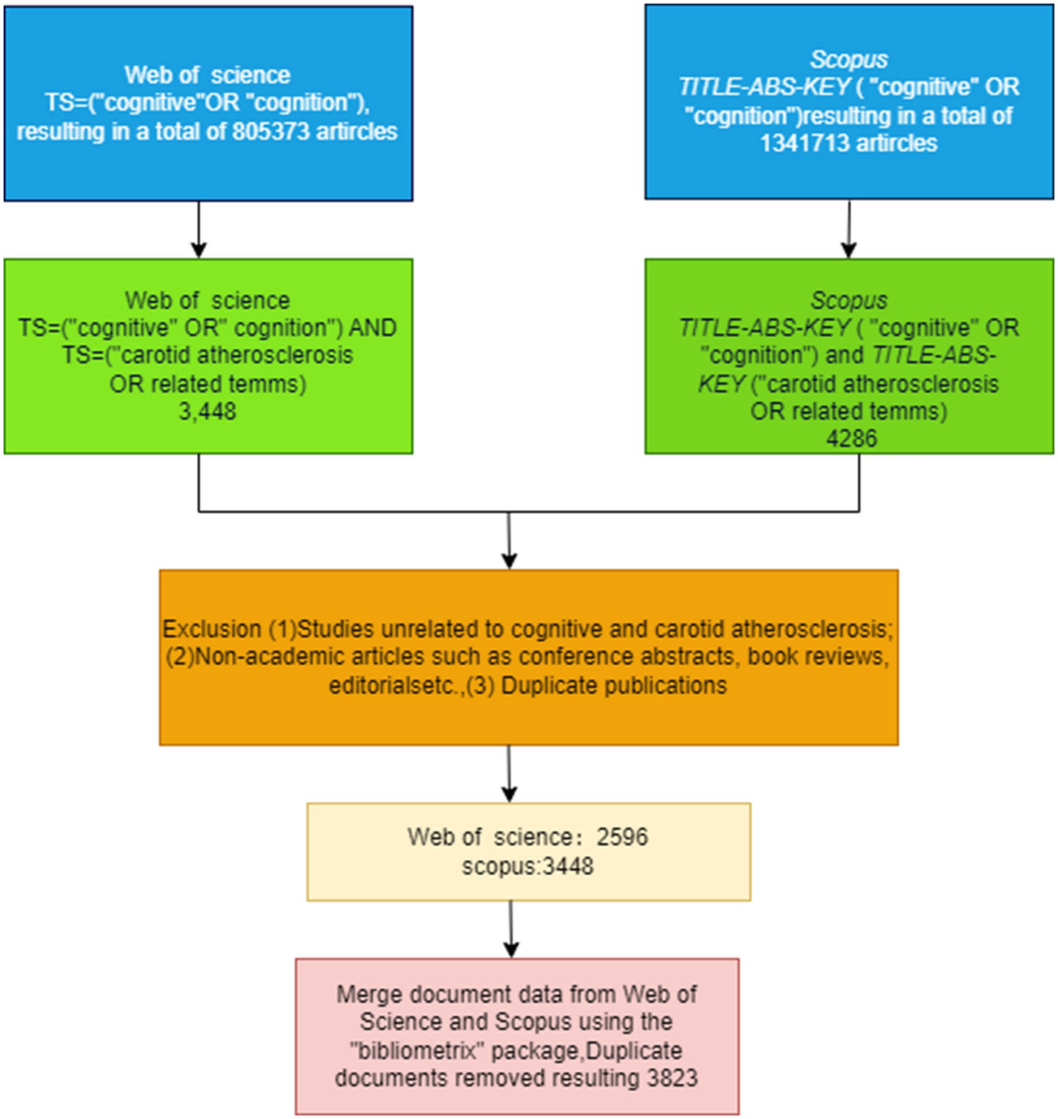
Flowchart of literature screening and data processing for bibliometric analysis.

This study retrieved English-language publications related to the intersection of cognitive function and carotid atherosclerosis from the Web of Science Core Collection (WoSCC) and Scopus databases. In WoSCC, the search strategy was defined as follows: *TS = (“cognitive” OR “cognition”) AND TS = (“carotid atherosclerosis” OR “carotid atheromatous” OR “carotid arteriosclerosis” OR “cervical atherosclerosis” OR “carotid arter” OR “carotid atherosclerotic plaque” OR “neck artery atherosclerosis”)**. An equivalent query was used in Scopus: *TITLE-ABS-KEY (“cognitive” OR “cognition”) AND TITLE-ABS-KEY (“carotid atherosclerosis” OR “carotid atheromatous” OR “carotid arteriosclerosis” OR “cervical atherosclerosis” OR “carotid arter” OR “carotid atherosclerotic plaque” OR “neck artery atherosclerosis”)**. The search was restricted to English articles and reviews published between 1995 and 2025. Exclusion criteria were: (1) studies unrelated to cognitive function or carotid atherosclerosis; (2) non-academic documents (e.g., conference abstracts, book reviews, editorials); (3) duplicate records. After initial screening, 2,596 records from WoSCC and 3,448 records from Scopus were retained. These datasets were merged and deduplicated using the bibliometrix package (v4.2.0) in R, resulting in 3,823 unique publications. Metadata exported for analysis included titles, authors, institutions, abstracts, author keywords, and cited references, saved in .txt and .csv formats. The cleaned dataset was subsequently imported into CiteSpace (v6.2. R1) and VOSviewer (v1.6.20) to examine publication trends, collaboration networks, keyword co-occurrence, and co-citation structures. The overall screening procedure is illustrated in Figure 1.

### 2.2 Literature screening and data preprocessing

Multiple bibliometric tools were employed to ensure methodological rigor and reproducibility. The analyses were conducted using R (v4.5.0) with the following major packages and software versions: bibliometrix (v4.2.0), CiteSpace (v6.4.1), VOSviewer (v1.6.19), and ggplot2 (v3.4.4). Each tool was used for its specific strengths within the analytical workflow.

Bibliometrix: calculated bibliometric indicators (e.g., publication counts, citation metrics, author productivity), generated temporal trend plots and collaboration networks, and provided quantitative evidence for research evolution (21). CiteSpace: conducted keyword and co-citation analyses. The time slice was set to 1 year, with the top 50 items retained in each slice. Pathfinder and sliced network pruning were applied, and burst detection was enabled to identify emerging topics (22, 23). ggplot2: optimized the visual presentation of results by unifying graphical styles and enhancing clarity. The deduplication workflow consisted of the following steps: (1) importing WoSCC and Scopus datasets; (2) performing triple matching based on DOI, title, and first author; (3) removing exact and partial duplicates; (4) manually verifying records to retain the earliest publication; (5) exporting the final unique dataset for analysis. The final curated corpus comprised 3,823 independent publications. To enhance transparency and reproducibility, all primary analytical settings and parameter configurations are detailed in Table 1 and the Methods section of the file Supplementary method.

**Table 1:** Top 10 countries or regions by Carotid Atherosclerosis and Cognitive publication volume.

Country	Articles %	SCP	MCP	MCP %
China	28.1	996	80	7.4
USA	18.7	613	101	14.1
Japan	7.9	280	21	7
Italy	4.1	130	25	16.1
Korea	3.9	133	16	10.7
UK	3.1	92	28	23.3
Netherlands	2.8	84	23	21.5
Germany	2.7	85	19	18.3
Canada	2.4	79	11	12.2
India	2.2	81	4	4.7

SCP, single country publications; MCP, multiple country publications.

## 3 Results

### 3.1 Publication trend over time

**Figure 2:**
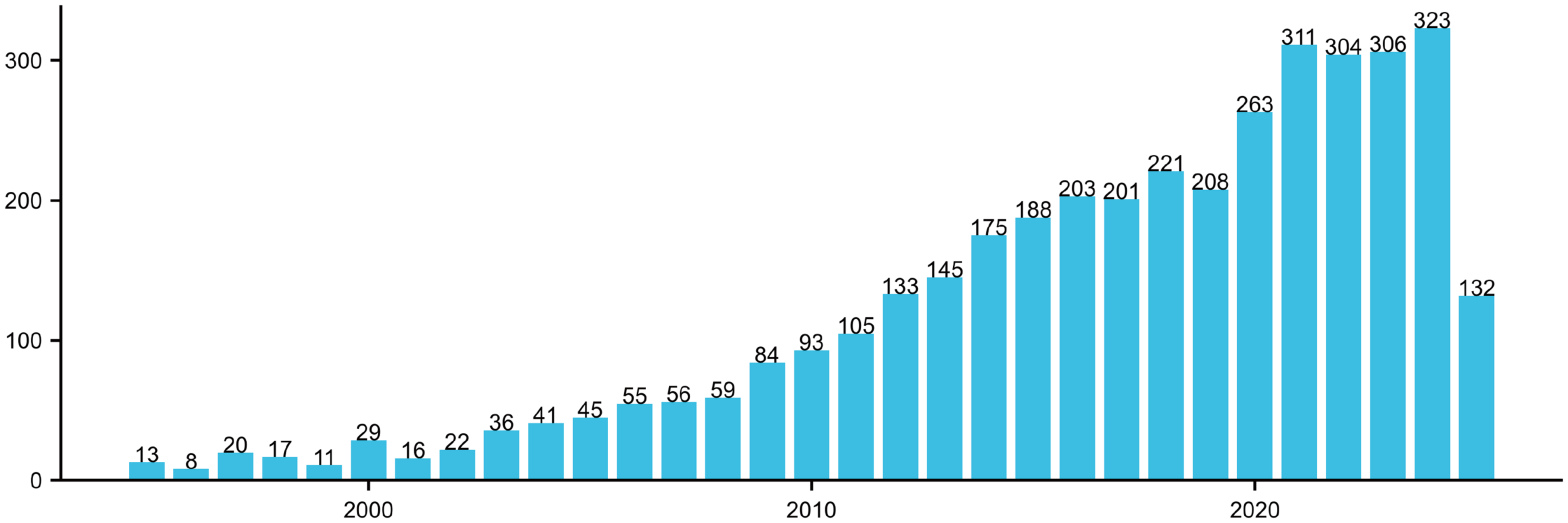
Annual publication trends in the field of carotid atherosclerosis and cognitive research (1995–2025).

Figure 2 illustrates the annual publication trend from 1995 to 2025 concerning the themes of cognitive function and carotid atherosclerosis, reflecting the evolutionary trajectory of this research domain. During the early stage (1995–2005), the number of publications was relatively low, averaging fewer than 50 articles per year, indicating limited academic attention and an exploratory phase. From 2006 onward, research output began to rise steadily, surpassing 100 publications annually by 2015, marking growing scholarly interest in this interdisciplinary area. A sharp increase was observed after 2016, with annual outputs stabilizing around 300 publications since 2020 and peaking at 323 in 2023. This trend suggests a heightened focus on the mechanistic links between cognitive impairment and cerebrovascular pathology, drawing attention from disciplines such as neuroscience, geriatrics, and public health. Notably, the number of publications recorded for 2025 (132 articles) is considerably lower, likely due to database updates lagging behind or the data not covering the full calendar year, and should therefore be interpreted with caution.

### 3.2 Distribution characteristics of the literature

**Figure 3:**
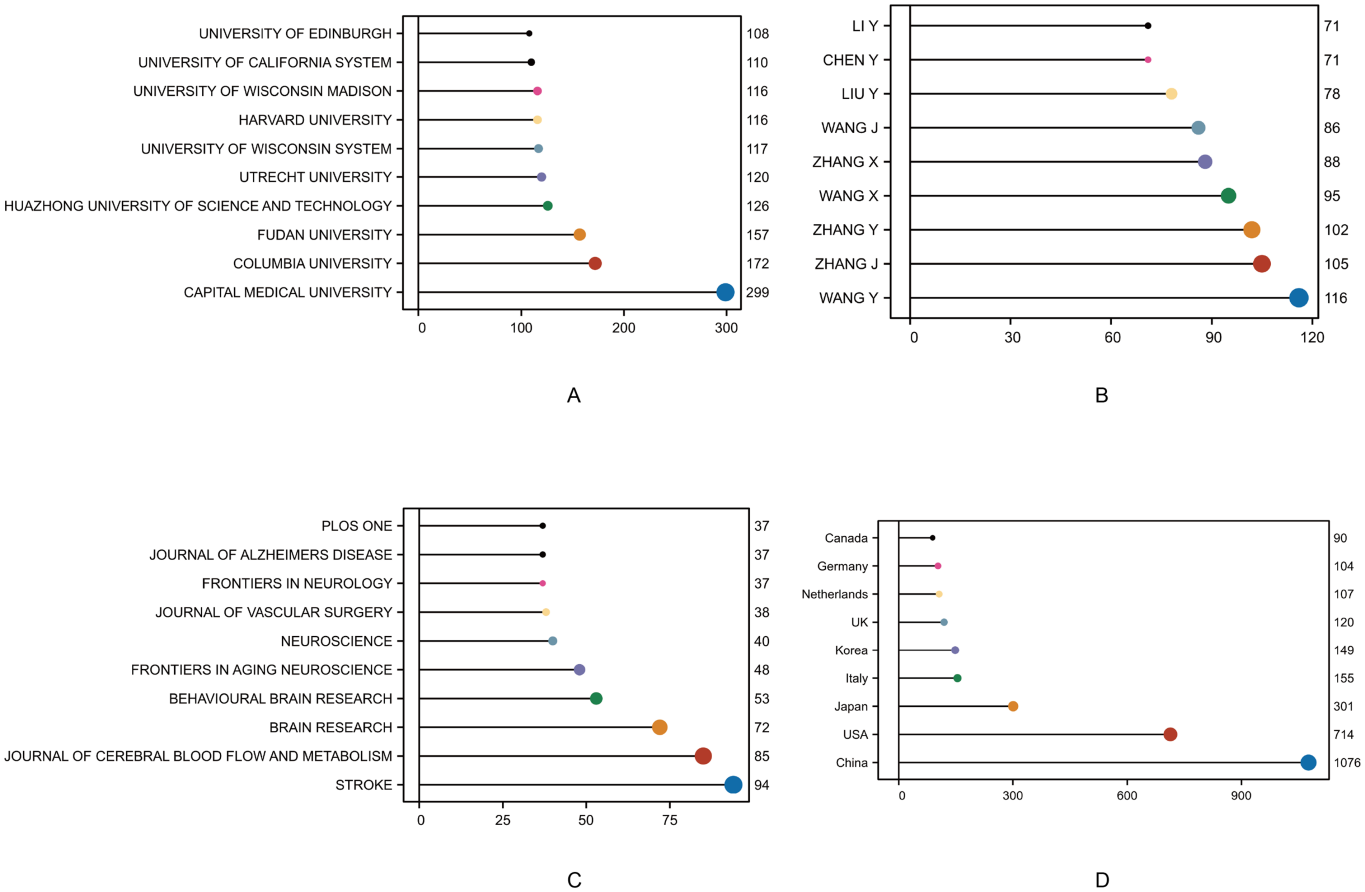
Top institutions, journals, articles, and authors in the field based on publication and citation metrics. **(A)** Top 10 institutions by number of publications. **(B)** Top 10 authors by number of published articles. **(C)** Top 10 journals by number of published articles. **(D)** Top 10 countries by number of published articles.

Figure 3 presents the distribution of publications across institutions, authors, journals, and countries. In terms of institutional productivity (Figure 3A), Capital Medical University ranked first with 299 publications, highlighting its significant influence in the field, followed by Columbia University (272 articles) and Fudan University (232 articles), alongside prominent institutions such as Harvard University and the University of Wisconsin system. Overall, leading research institutions from China and the United States dominate the field, reflecting both countries’ sustained investment in research on cognitive impairment and cerebrovascular diseases. Author productivity (Figure 3B) further underscores the active involvement of Chinese scholars, with authors such as WANG Y, ZHANG Y, TANG Y, and YAN Y each contributing over 100 publications, indicating high research output and strong collaborative capacity, primarily among Chinese academic institutions. Regarding journal distribution (Figure 3C), publications were primarily concentrated in high-impact journals focusing on neuroscience and cerebrovascular disease, such as Stroke (84 articles), Journal of Cerebral Blood Flow and Metabolism (65 articles), and Brain Research (72 articles). Journals related to neurodegenerative conditions in aging, including Frontiers in Aging Neuroscience and Journal of Alzheimer’s Disease, were also among the top outlets, suggesting a close linkage between this research field and diseases such as Alzheimer’s and stroke, with substantial clinical translational value. In terms of national contributions (Figure 3D), China ranked first with 1,016 publications, followed by the United States (714 articles). Other countries with notable outputs included Japan, South Korea, the United Kingdom, Germany, the Netherlands, and Canada, demonstrating the formation of a globally distributed research network centered around China and the United States, with strong potential for international collaboration.

### 3.3 Country distribution and international collaboration

**Figure 4:**
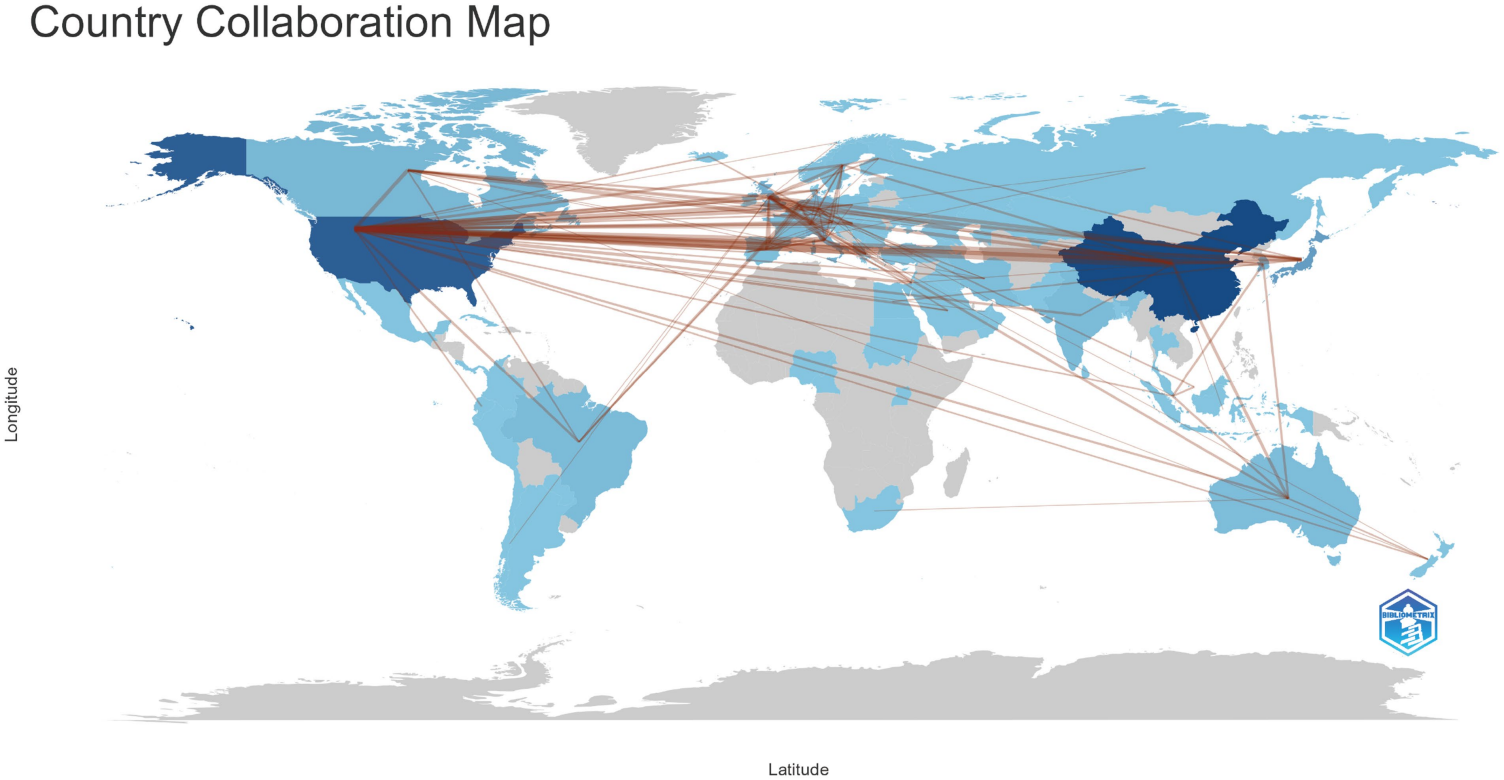
The collaboration between countries and regions on Carotid Atherosclerosis and Cognitive Research. The color segmentation includes blue (with publications) and gray (without publications). The thickness of the red lines indicates the number of co-published papers. The color intensity corresponds to the number of publications.

Table 1 summarizes the scientific contributions of the major publishing countries. China is the leading contributor in this field, accounting for 28.1% of all publications, with 996 single-country publications (SCP) and 80 multi-country publications (MCP), yielding an international collaboration rate of 7.4%. The United States ranks second, contributing 18.7% of the total output and demonstrating a higher level of international collaboration (MCP = 101; 14.1%), indicating stronger global engagement. Japan accounts for 7.9% of total publications, with an international collaboration rate of approximately 7%. Among European countries, Italy (16.1%), the United Kingdom (23.3%), the Netherlands (21.5%), and Germany (18.3%) show relatively high MCP rates, reflecting the strong interconnectedness of European research networks. In contrast, India exhibits the lowest international collaboration rate at 4.7%. Figure 4 illustrates the global collaboration network among countries, where denser connecting lines indicate stronger research partnerships. The results show that the United States occupies a central position in the international collaboration network, maintaining extensive academic links with China, the United Kingdom, Germany, Canada, and Japan. Additionally, European countries such as the United Kingdom, Germany, the Netherlands, and Italy form a tightly knit regional collaboration cluster, highlighting a Europe- and North America-centered global scientific cooperation structure. For clearer visualization, an accompanying table (Supplementary Table S2) is provided within Figure 4 to further illustrate the strength and frequency of collaborations among the major countries.

### 3.4 Analysis of globally highly cited publications

Table 2 summarizes the 10 most globally cited publications in this field. These influential works were primarily published in high-impact journals, including European Heart Journal, Journal of Hypertension, Cerebrovascular Diseases, Stroke, The Lancet, Diabetes Care, JAMA, International Journal of Epidemiology, and the European Journal of Vascular and Endovascular Surgery. The most highly cited article is the 2013 hypertension management guideline by Mancia et al. (24) (4,045 citations). This is followed by the 2018 updated hypertension guideline by Williams et al. (25) (2,300 citations) and the 2008 guideline on the management of ischemic stroke and transient ischemic attack by Ringleb et al. (26) (2,285 citations). Other highly cited publications include Pantoni and Garcia (27) seminal review on the pathogenesis of leukoaraiosis, Hankey’s (61) stroke review published in The Lancet, Silverstein et al.’s (28) guideline for the care of children and adolescents with type 1 diabetes, Fried et al.’s (29) study on mortality risk among older adults, Völzke’s (62) cohort profile of the Study of Health in Pomerania, Naylor et al.’s (30) guideline on carotid and vertebral artery disease, and Yaffe’s (63) investigation of estrogen therapy and cognitive function. Overall, these publications span from 1997 to 2018 and encompass three major categories: clinical practice guidelines, narrative reviews, and cohort studies.

**Table 2:** Top 10 articles with the highest global citations.

Number	First Author	Article name	Journal name	Year	Global citations
1	Giuseppe Mancia	2013 ESH/ESC guidelines for the management of arterial hypertension: the Task Force for the Management of Arterial Hypertension of the European Society of Hypertension (ESH) and of the European Society of Cardiology (ESC)	European Heart Journal	2013	4,045
2	Bryan Williams	2018 ESC/ESH Guidelines for the management of arterial hypertension: The Task Force for the management of arterial hypertension of the European Society of Cardiology and the European Society of Hypertension: The Task Force for the management of arterial hypertension of the European Society of Cardiology and the European Society of Hypertension	Journal Of Hypertension	2018	2,300
3	Peter A Ringleb	Guidelines for management of ischaemic stroke and transient ischaemic attack 2008	Cerebrovascular Diseases	2008	2,285
4	L Pantoni	Pathogenesis of leukoaraiosis: a review	Stroke	1997	1,053
5	Graeme J Hankey	Stroke	Lancet	2017	1,007
6	Janet Silverstein	Care of children and adolescents with type 1 diabetes: a statement of the American Diabetes Association	Diabetes Care	2005	931
7	L P Fried	Risk factors for 5-year mortality in older adults: the Cardiovascular Health Study	JAMA	1998	887
8	Henry Völzke	Cohort profile: the study of health in Pomerania	International Journal of Epidemiology	2011	877
9	A R Naylor, J-B Ricco	Editor’s Choice - Management of Atherosclerotic Carotid and Vertebral Artery Disease: 2017 Clinical Practice Guidelines of the European Society for Vascular Surgery (ESVS)	European Journal of Vascular and Endovascular Surgery	2018	863
10	K Yaffe	Estrogen therapy in postmenopausal women: effects on cognitive function and dementia	JAMA	1998	740

### 3.5 Keyword network, clustering, and burst detection analysis

**Figure 5:**
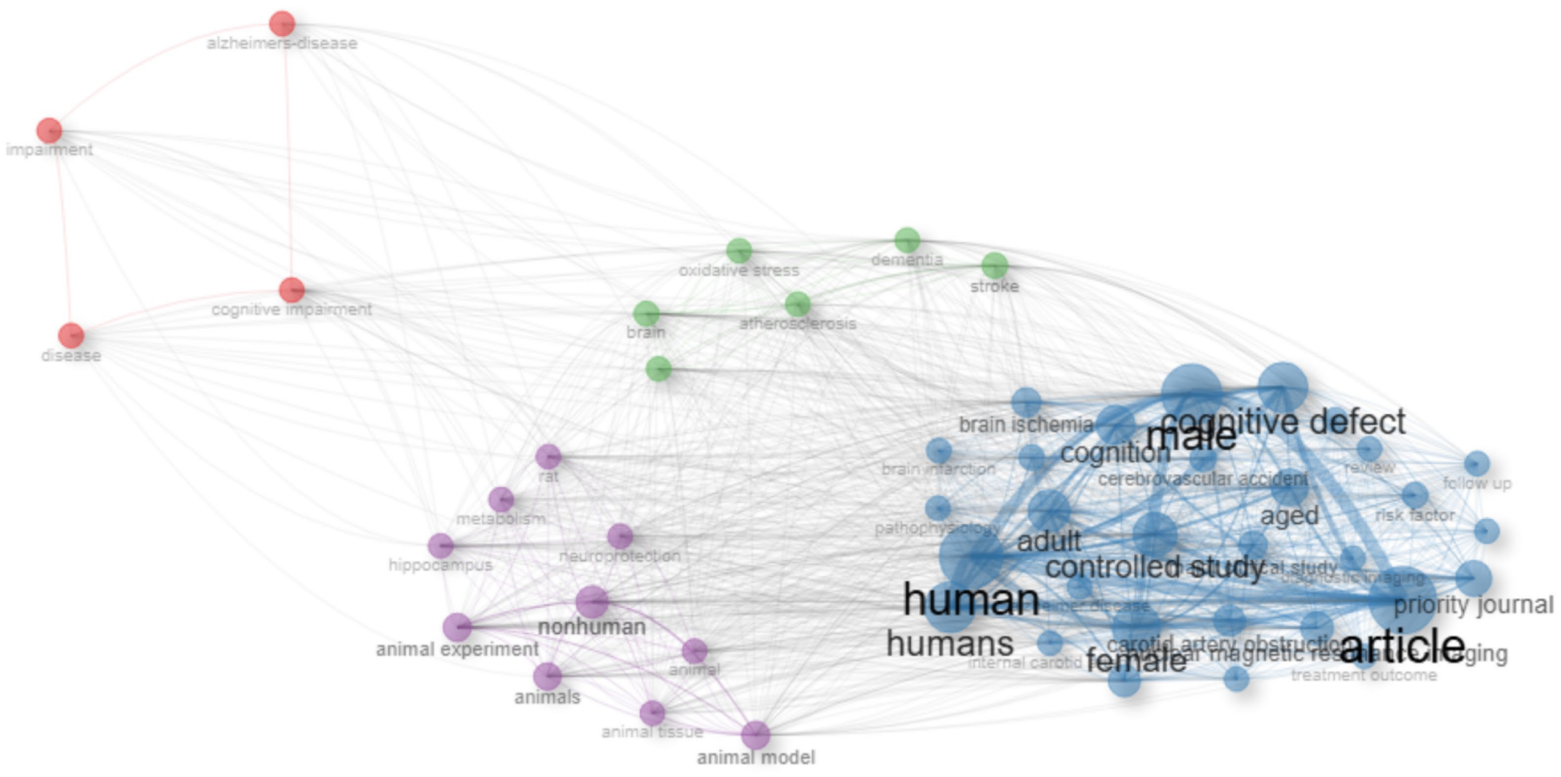
Keyword co-occurrence network generated using the R package bibliometrix. Node size represents keyword frequency, line thickness reflects co-occurrence strength, and colors indicate different thematic clusters.

**Figure 6:**
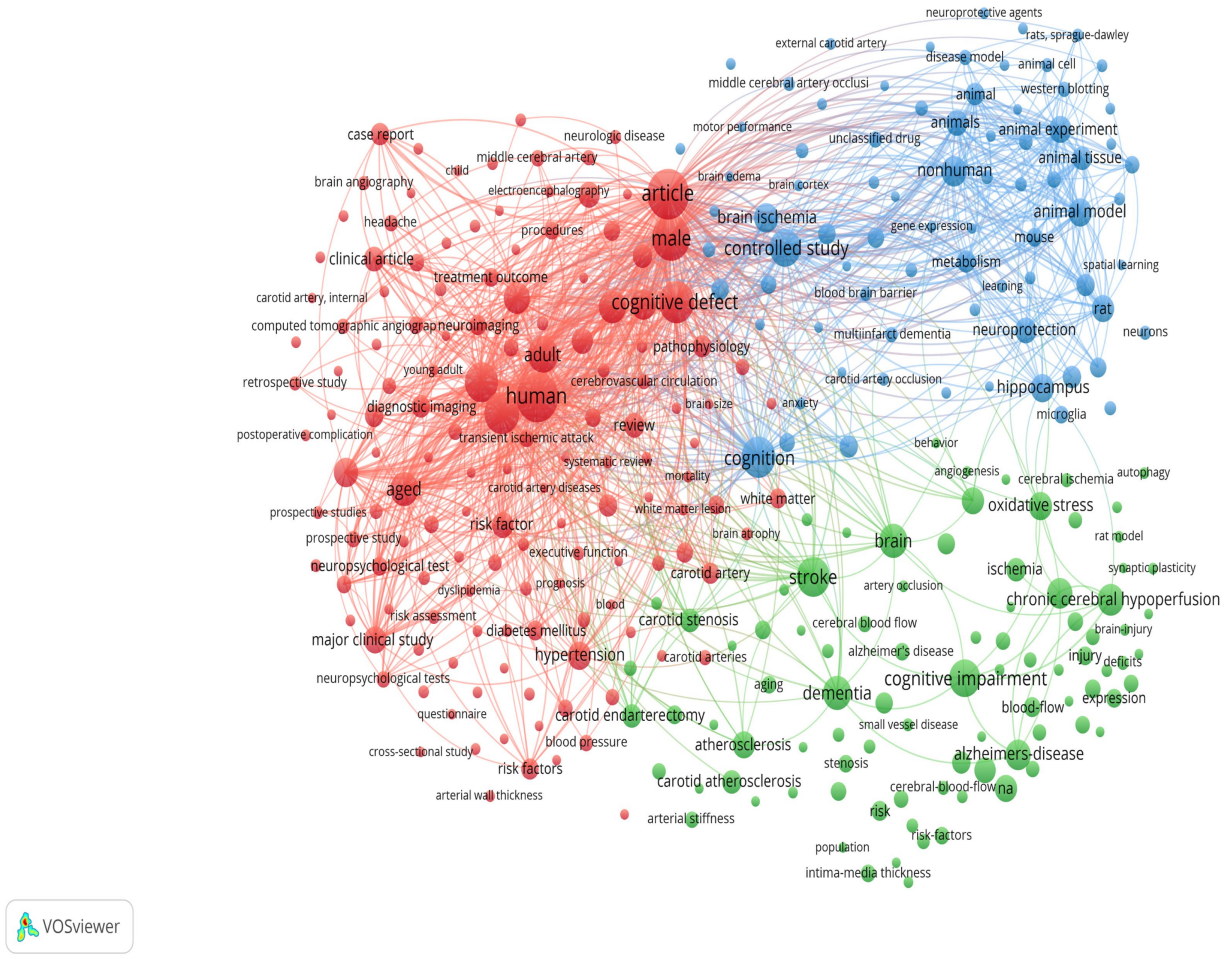
Clustered keyword co-occurrence network generated using VOSviewer.

**Figure 7:**
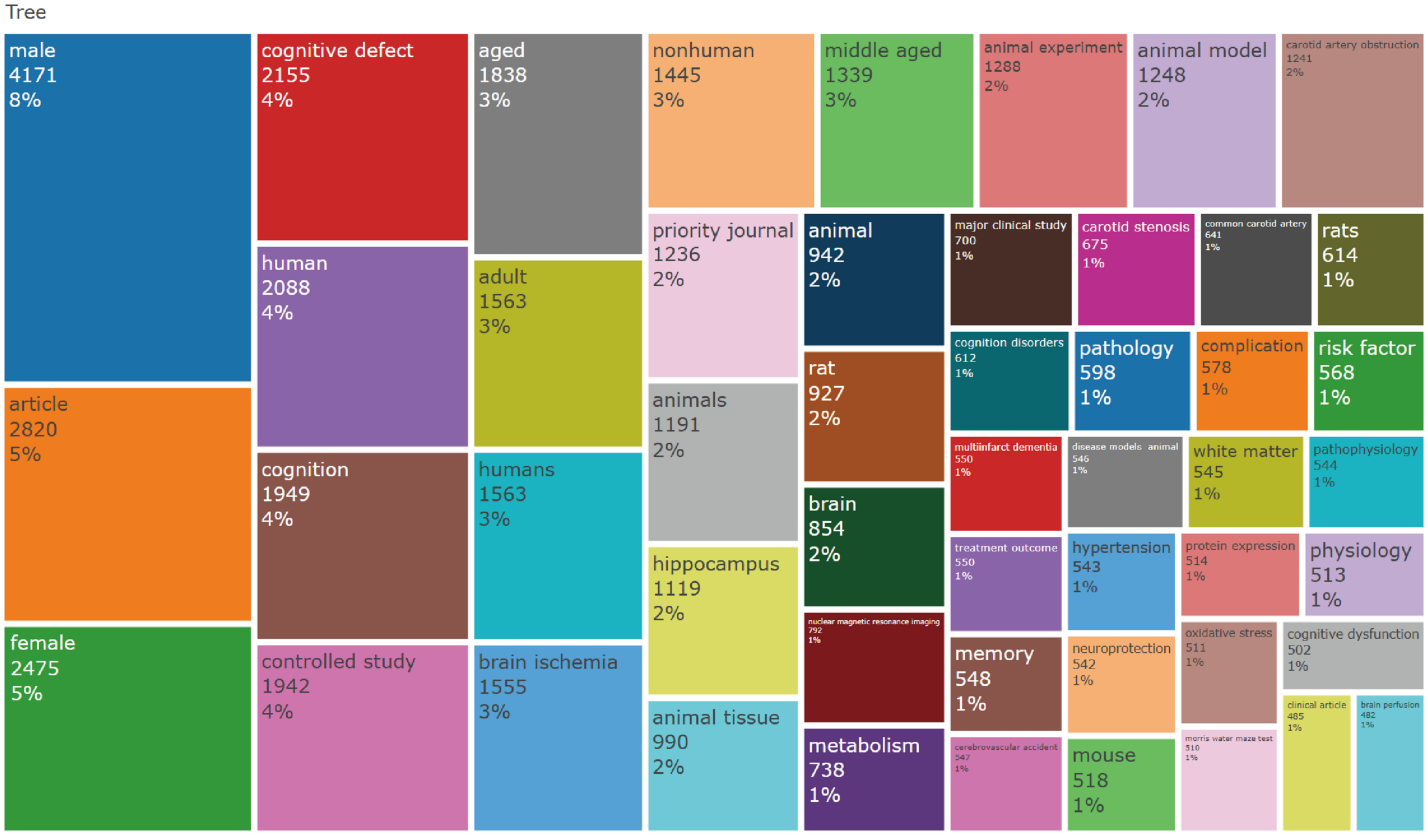
Keyword TreeMap generated using the R package bibliometrix.

**Figure 8:**
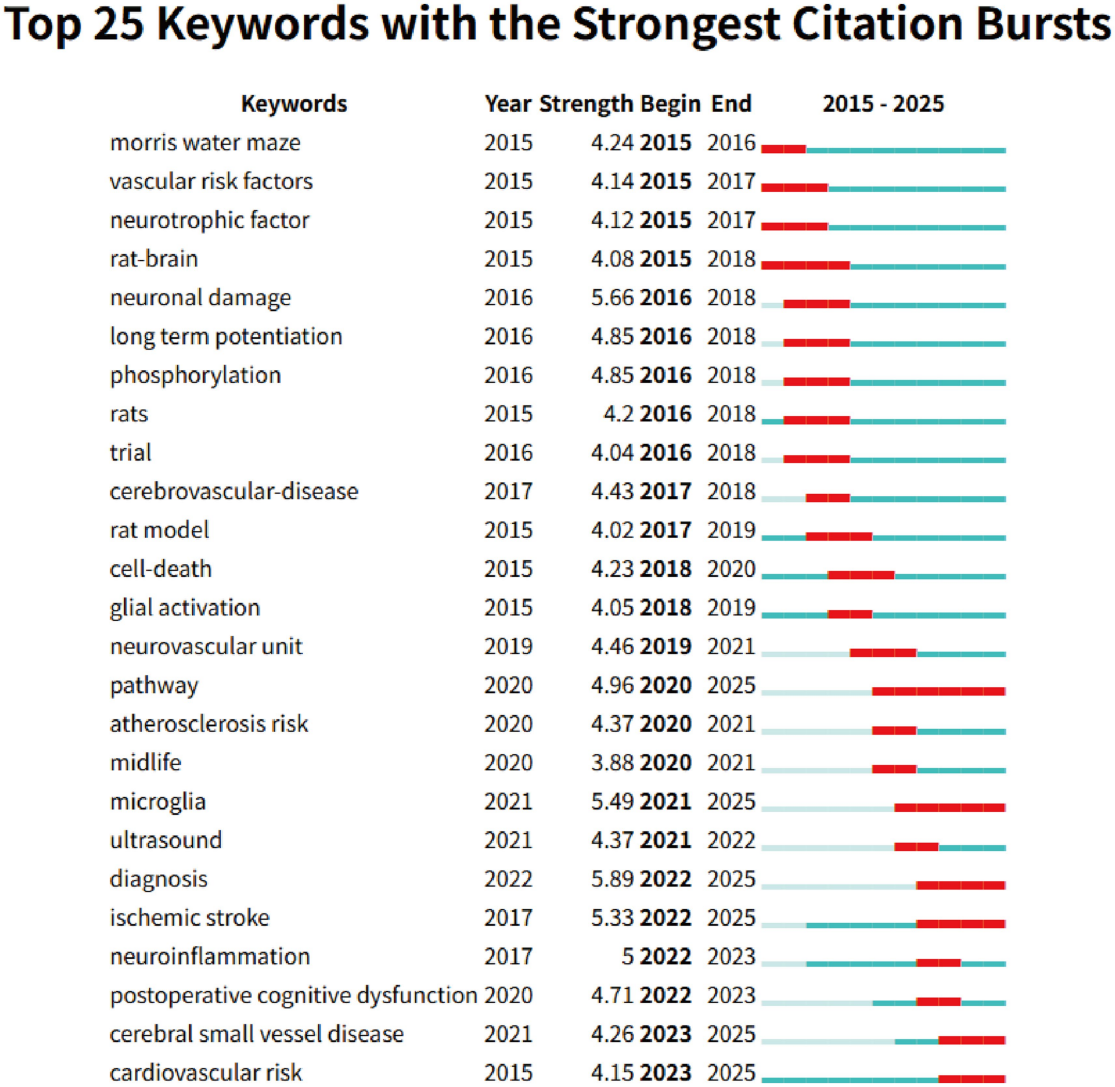
Top 25 keyword bursts identified using CiteSpace.

The keyword co-occurrence analysis revealed the overall thematic structure of research on carotid atherosclerosis and cognitive function. As shown in Figure 5, the co-occurrence network generated using bibliometrix can be divided into four major clusters: the blue cluster, representing population-based studies and cognitive impairment; the purple cluster, corresponding to animal experiments and model research; the green cluster, focusing on neural mechanisms and metabolic regulation; and the red cluster, associated with Alzheimer’s disease and neurodegenerative mechanisms. Together, these clusters outline a multidimensional and cross-disciplinary framework that characterizes the field. Figure 6 further presents the keyword co-occurrence network constructed using VOSviewer, demonstrating a clustering pattern highly consistent with that in Figure 5. The red cluster primarily contains clinical and epidemiological terms (e.g., human, male, controlled study); the green cluster highlights key pathological mechanisms such as oxidative stress, inflammation, and endothelial dysfunction; while the blue cluster centers on structures and conditions related to neurodegeneration, including the hippocampus, brain ischemia, and Alzheimer’s disease. Taken together, these patterns indicate a transition of research focus from clinical characterization toward deeper exploration of biological mechanisms, reflecting a shift from macro-level observations to micro-level mechanistic investigations. The keyword tree map (Figure 7) further illustrates the distribution of high-frequency terms. Among them, male (8%) and female (5%) were the most prominent, underscoring the foundational role of demographic characteristics in this research domain. Other frequently occurring keywords—such as cognitive defect, controlled study, and brain ischemia—highlight the dominant themes of cognitive assessment and cerebrovascular pathology throughout the literature. The burst-detection analysis (Figure 8) captured the temporal evolution of research hotspots. Earlier bursts, including morris water maze, neuronal damage, and long-term potentiation, were mainly associated with fundamental neurobiological mechanisms. In contrast, more recent bursts—such as microglia, ultrasound, diagnosis, postoperative cognitive dysfunction, and cerebral small vessel disease—indicate a progressive shift toward precision neuroimaging, postoperative cognitive assessment, and vascular-related clinical applications. Overall, the field demonstrates a clear trend from foundational mechanistic inquiry toward clinically oriented identification and intervention strategies.

## 4 Discussion

Drawing upon data retrieved from the Web of Science Core Collection and Scopus databases, this study undertakes a comprehensive bibliometric analysis of the interdisciplinary domain of cognitive function and carotid atherosclerosis spanning the period from 1995 to 2025. By examining publication trends, leading research entities, patterns of international collaboration, highly cited references, and the temporal evolution of keywords, the study delineates the structural development and thematic foci of the field. Analysis of annual publication output reveals a clear trajectory from an exploratory phase to a period of accelerated growth, with a marked surge in research activity observed since 2016 and a peak reached in 2023. This upward trend likely reflects increasing scholarly recognition of the pathophysiological link between carotid atherosclerotic burden and cognitive impairment. Furthermore, advances in interventional approaches—such as carotid artery stenting (CAS) and carotid endarterectomy—have contributed to improved cerebral perfusion and cognitive outcomes in clinical populations, thereby providing empirical impetus for the expanding body of literature in this area (31, 32). The evolution of keywords further illustrates that the increase in publications reflects not only an expansion in research output, but also a thematic shift from hemodynamic mechanisms toward a more integrative understanding of the vascular–immune–neural pathways involved in disease progression. This transition indicates that research in this field is increasingly converging on biological mechanisms with clear translational potential, highlighting emerging targets that bridge basic pathology and clinical intervention. In terms of publication output, China and the United States have demonstrated clear leadership within this research domain. Chinese research institutions—such as Capital Medical University and Fudan University—along with prolific authors including WANG Y and ZHANG Y, exhibit high levels of scientific productivity, reflecting China’s rapid advancement in the intersection of cognitive impairment and cerebrovascular disease. Concurrently, the United States occupies a central position in international collaboration networks, maintaining extensive partnerships with multiple countries, thereby underscoring its pivotal role as a global hub in the scientific landscape. European countries, notably the United Kingdom, Germany, and Italy, also display a high degree of cross-national collaboration, indicative of a broader trend toward international integration, resource sharing, and collective expertise. These patterns are likely shaped by differing levels of national investment in scientific research and healthcare infrastructure (33, 34). Given that China is projected to have the largest aging population globally in the coming decades, this demographic shift may partly account for the growing volume of research focused on cognition and cognitive impairment within the country (4, 35). In terms of journal distribution, leading neuroscience journals such as Stroke and Brain Research have served as primary publication venues, indicating a high degree of specialization and concentration in scholarly output. From the perspective of international collaboration, although China ranks first in total publication volume, its proportion of internationally co-authored papers remains relatively low, suggesting that research activities are predominantly domestically oriented. In contrast, several European countries—particularly the United Kingdom, Germany, and the Netherlands—exhibit significantly higher levels of international collaboration. The United Kingdom, with an international collaboration rate of 23.3%, exemplifies strong openness and connectivity within the global academic network. The United States, as the most active country in international research partnerships, has established robust academic ties with numerous nations, especially China, the Netherlands, and Japan, thereby reinforcing its central role within the global scientific ecosystem. The evolution of keywords further illustrates that the increase in publications reflects not only an expansion in research output, but also a thematic shift from hemodynamic mechanisms toward a more integrative understanding of the vascular–immune–neural pathways involved in disease progression. This transition indicates that research in this field is increasingly converging on biological mechanisms with clear translational potential, highlighting emerging targets that bridge basic pathology and clinical intervention. These differences in international collaboration may be shaped by variations in research policies, funding mechanisms, and the structural organization of national scientific systems. For example, the European Union has long promoted cross-border collaboration through large-scale research frameworks such as Horizon 2020, which structurally encourage multinational participation. In contrast, many of China’s large multi-center studies are predominantly led by domestic institutions, a system-level characteristic that may naturally reduce the proportion of multinational collaborations (35, 36).

Analysis of the top 10 most cited publications shows that five are clinical practice guidelines, two are cohort studies, and three are review articles. This distribution highlights the field’s reliance on three major categories of evidence: authoritative guidelines, comprehensive reviews, and large-scale longitudinal studies. Clinical guidelines, in particular, not only offer standardized recommendations for the prevention and management of hypertension, atherosclerosis, and other cerebrovascular diseases, but also provide an essential clinical framework for understanding the association between vascular factors and cognitive function. The most cited publication is the 2013 ESH/ESC Guidelines for the Management of Arterial Hypertension by Giuseppe Mancia et al. (24), published in European Heart Journal, which outlines diagnostic criteria, risk stratification, antihypertensive therapy, and lifestyle modifications, emphasizing comprehensive assessment and individualized management. The second most cited publication is the 2018 ESC/ESH updated hypertension guideline by Williams et al. (25), published in Journal of Hypertension, which further refines blood pressure targets, underscores early intervention and combination therapy, and recommends maintaining systolic blood pressure at 120–129 mmHg for high-risk individuals, reflecting a more proactive management approach. The prominence of these guidelines underscores the role of hypertension and carotid atherosclerosis as major risk factors for cognitive decline and explains the strong influence of guideline literature in this field (25). Ranked third is the 2008 Guidelines for the Management of Ischemic Stroke and Transient Ischemic Attack by Ringleb et al. (26), published in Cerebrovascular Diseases. This document provides detailed recommendations on imaging-based diagnosis, thrombolysis, antiplatelet and anticoagulation therapy, secondary prevention, and rehabilitation, emphasizing the importance of identifying high-risk patients and strategies to prevent post-stroke cognitive impairment. The top-cited list also includes guidelines related to metabolic disorders, such as the American Diabetes Association’s guideline for the care of children and adolescents with type 1 diabetes by Janet Silverstein et al. (28) (published in Diabetes Care), which highlights glycemic management, complication screening, and multidisciplinary support. The guideline also notes that glycemic fluctuations and recurrent hypoglycemia may impair cognitive development, and that some adolescents show increased carotid intima–media thickness (IMT), suggesting early vascular pathology (28). Another influential document is the 2017 ESVS Clinical Practice Guideline by Naylor et al. (30), published in the European Journal of Vascular and Endovascular Surgery, which covers screening, diagnosis, risk stratification, and treatment for carotid and vertebral artery disease and provides important clinical insights into the links among asymptomatic carotid stenosis, cerebral hypoperfusion, and mild cognitive impairment. Among the review articles, the classic review by Pantoni and Garcia (27) in Stroke proposes that chronic cerebral hypoperfusion may underlie leukoaraiosis, emphasizing the close relationship between microvascular pathology and cognitive decline and establishing a theoretical basis for subsequent imaging and experimental studies (27). In addition, several highly cited cohort studies provide robust evidence on the associations among cognitive decline, mortality risk, and multiple clinical factors, contributing to risk stratification and prevention strategies for vascular cognitive impairment (29). Collectively, these influential publications indicate a shift from descriptive observations of comorbidity toward a more integrated framework linking mechanisms, risk stratification, and intervention pathways. However, their thematic scope remains fragmented, with most focusing on hypertension, stroke, metabolic disease, or white matter pathology rather than systematically depicting the specific intersection of carotid atherosclerosis × cognitive function. The present study addresses this gap by applying bibliometric methods to comprehensively map the knowledge structure, thematic evolution, and collaborative networks within this interdisciplinary domain.

To facilitate the interpretation of keyword clusters, the following section independently summarizes relevant biological mechanisms and disease pathways reported in the existing literature, providing contextual background for the bibliometric findings. Overall, keyword co-occurrence and clustering analyses indicate that research at the intersection of cognitive function and carotid atherosclerosis primarily converges on three thematic domains: (1) clinical assessment and epidemiological characteristics; (2) overlapping mechanisms of neurodegenerative diseases, Alzheimer’s disease, and vascular cognitive impairment; (3) inflammation- and oxidative stress-mediated cellular injury pathways. Notably, a close association has been observed between carotid pathology and Alzheimer’s disease (AD). Numerous studies have reported that carotid atherosclerosis is strongly linked to an increased risk of AD. Systematic reviews and meta-analyses have shown that in Asian populations, factors such as frailty, carotid atherosclerosis, hypertension, low diastolic blood pressure, and type 2 diabetes significantly elevate the risk of developing AD (37–39). Experimental evidence further supports this connection: chronic cerebral hypoperfusion induced by carotid artery ligation has been widely used as an established model for investigating AD-related mechanisms (40–42). From a neuropathological perspective, carotid atherosclerosis affects cognitive function through multiple interconnected pathways. First, plaque instability can lead to the release of embolic fragments, causing microemboli and multifocal microinfarctions. Once these emboli travel into cerebral vessels, they trigger localized ischemia, resulting in cognitive impairment, often occurring concurrently with ischemic events (43). Neuroimaging studies have also demonstrated that plaque pulsatility and strain parameters are closely associated with vascular cognitive decline and are accompanied by increases in white matter hyperintensities (WMH) and subclinical microemboli (44). Second, cerebral hypoperfusion represents another key mechanism. Common causes include carotid stenosis, intracranial atherosclerosis, heart failure, and other hemodynamic abnormalities. Chronic hypoperfusion can disrupt neuronal metabolism and compromise white matter integrity, ultimately leading to WMH, cortical atrophy, and deficits in attention, executive function, and memory (45). Third, hypoperfusion and recurrent microembolization can induce neuroinflammation, resulting in blood–brain barrier disruption, overactivation of microglia and astrocytes, and persistent release of pro-inflammatory cytokines (e.g., IL-1β, TNF-*α*). These processes promote neuronal apoptosis and synaptic degeneration, accelerating cognitive decline (46–48). Animal studies have shown that chronic carotid ligation leads to activation of the NLRP3 inflammasome, increased reactive oxygen species (ROS) production, and thickening of the microvascular basement membrane—all of which are strongly associated with cognitive impairment (49). Animal studies have shown that chronic carotid ligation leads to activation of the NLRP3 inflammasome, increased reactive oxygen species (ROS) production, and thickening of the microvascular basement membrane—all of which are strongly associated with cognitive impairment (50, 51). These multi-layered mechanisms collectively explain why early cognitive decline and aberrant brain network connectivity are frequently observed even in asymptomatic individuals with carotid stenosis. Substantial evidence supports the links between cerebral hypoperfusion, vascular dementia, neuroinflammation, oxidative stress, and cognitive deterioration. For example, Lin et al. (52) demonstrated that bilateral carotid artery ligation disrupts the blood–brain barrier, promotes T-cell and neutrophil infiltration, and exacerbates inflammation, whereas suppression of neuroinflammation significantly improves cognitive outcomes. Additional studies have shown that chronic cerebral hypoperfusion induces sequential activation of inflammasomes (e.g., NLRP3) and their downstream cytokines IL-1β and IL-18, triggering apoptosis and pyroptosis pathways, glial activation, white matter lesions, and hippocampal neuronal loss (53–56). Most recently, a prospective cohort study from the PESA project by Tristao-Pereira et al. (57), involving 4,184 asymptomatic individuals aged 40–54 years, found that those at high cardiovascular risk exhibited significantly accelerated reductions in cortical [18F]FDG uptake (*β* = −0.008 [95% CI –0.013 to −0.002]; pFDR = 0.040). Plasma neurofilament light chain, a biomarker of neurodegeneration, mediated approximately 20% of this association (*β* = 0.198 [0.008–0.740]; pFDR = 0.050). Furthermore, the progression of subclinical carotid atherosclerosis was independently associated with reduced 18F-fluorodeoxyglucose uptake in AD-related brain regions (*β* = −0.269 [95% CI –0.509 to −0.027]; *p* = 0.029) (57). Keyword Burst Analysis clearly illustrates the evolution of research hotspots. Early research focused on animal behavior and neuronal damage, transitioning to a mid-phase emphasis on neurovascular coupling and cell death mechanisms, and more recently, terms like “microglia,” “ischemic stroke,” and “postoperative cognitive dysfunction” have consistently emerged. This shift indicates that research has gradually expanded from basic mechanisms to more clinically relevant topics, such as interventions for cognitive impairment and disease diagnosis. Increasing evidence suggests that microglia play a crucial role in the relationship between carotid artery disease and cognitive impairment. For instance, Zhao et al. (58) employed bilateral common carotid artery occlusion (BCCAO) to establish a Sprague–Dawley rat model of chronic cerebral hypoperfusion (CCH). Their findings revealed that mitochondrial damage and increased ROS levels in the rat brain, alongside reduced overexpression of Sirt1 in glial cells, were reversed with NAD + treatment, resulting in improved cognitive function in the rats (58). Moreover, treatments such as ketone bodies and acupuncture have been shown to alleviate cognitive impairment caused by bilateral carotid artery occlusion by modulating glial cell inflammation (59, 60). This progression, from model development to the exploration of disease mechanisms, provides important directions for future research, particularly regarding the continuous exploration of topics like postoperative cognitive dysfunction, cerebral small vessel disease, and neuroinflammation, which are likely to become research frontiers in the coming years. Accordingly, a growing body of mechanistic evidence supports an integrated immune–metabolic–neural network framework, providing a theoretical basis for future target-based cognitive protection strategies, such as inflammasome inhibition and glial cell modulation.

Despite the systematic review of the field’s development trends and research hotspots through bibliometric and visualization analysis, this study has several limitations. First, the data sources were limited to the Web of Science Core Collection and Scopus databases. Although the use of two major databases enhances coverage, relevant high-quality studies indexed in other databases—such as PubMed or CNKI—may have been omitted, potentially limiting the comprehensiveness of the findings. Second, the keyword analysis relies primarily on author-provided keywords and automatic extraction, which may result in insufficient merging of synonyms and inconsistencies in terminology standardization, potentially leading to biases in theme clustering. Additionally, the burst analysis in this study was conducted within a specific time window, meaning that high-quality studies that have not yet received widespread citation may not be fully represented, leading to temporal lag. Finally, bibliometric methods emphasize quantitative statistics and do not provide a detailed evaluation of the depth or methodological quality of the research, thus unable to replace traditional systematic reviews or meta-analyses in providing in-depth interpretations. These limitations highlight the need for future bibliometric studies to place greater emphasis on diversifying data sources, standardizing terminology, and integrating findings more closely with mechanistic and clinical research. Such approaches will enable a more comprehensive and accurate reflection of the true progress in cognitive research related to carotid atherosclerosis.

## 5 Conclusion

This study systematically delineates the evolution and emerging trends in research on carotid pathology and cognitive impairment, revealing both the steady accumulation of knowledge since the 1990s and the marked acceleration of scientific output since 2016. This rapid growth has been driven largely by the increasing integration of aging research, neurodegenerative disorders, and cerebrovascular science. Overall, research on carotid atherosclerosis–related cognitive impairment is transitioning from single-mechanism exploration toward a more integrated and translational framework. Future studies should align more closely with clinical needs in neurology and focus on several key directions: (1) multimodal neuroimaging combined with biomarker profiling to enhance early prediction and dynamic monitoring of cognitive decline; (2) long-term follow-up of carotid revascularization and interventional procedures to clarify the sustained cognitive benefits conferred by improved cerebral hemodynamics; (3) neurovascular-unit protection and repair strategies, including endothelial modulation, anti-inflammatory therapies, and oxidative-stress interventions, to slow neurodegenerative progression. Through deeper integration of mechanistic discovery and clinical application, future work may achieve breakthroughs in the neurovascular-immune mechanisms underlying chronic cerebral hypoperfusion, promoting the translation of basic research into precise interventions for vascular-related cognitive decline.

## Data Availability

The original contributions presented in the study are included in the article/Supplementary material, further inquiries can be directed to the corresponding authors.
