# Supplementary material for: A bibliometric analysis of research on cognitive function and carotid atherosclerosis: global trends and hotspots
Source: Front Neurol. 2026 Jan 13;16:1644172. doi: 10.3389/fneur.2025.1644172 (PMC12836883; doi:10.3389/fneur.2025.1644172)
Supplement: Supplementary file 2 [file Table_2.DOCX]

### ****STable 1. Summary of analytical tools and parameter settings****

| **Analytical Tool** | **Version** | **Function Module** | **Main Parameter Settings** |
| --- | --- | --- | --- |
| **bibliometrix (R)** | v4.2.0 | Annual publication trends, collaboration network, and productivity analysis | Default settings; yearSlice = 1995–2025 |
| **CiteSpace** | v6.4.1 | Co-citation and burst detection analysis | Time slice = 1 year; TopN = 50; Pruning = Pathfinder |
| **VOSviewer** | v1.6.19 | Clustering and co-occurrence visualization | Minimum occurrence = 5; Normalization = Association strength |
| **ggplot2 (R)** | v3.4.4 | Graphical visualization enhancement | Customized themes and color schemes |
| **Operating System** | Windows 11 (64-bit) | — | — |

**STable 2.Collaboration_WorldMap_bibliometrix**

|  | | |
| --- | --- | --- |
| **From** | **To** | **Frequency** |
| AUSTRALIA | NEW ZEALAND | 3 |
| AUSTRALIA | SINGAPORE | 5 |
| AUSTRALIA | SOUTH AFRICA | 2 |
| AUSTRIA | ALBANIA | 1 |
| AUSTRIA | LITHUANIA | 1 |
| AUSTRIA | MACEDONIA | 1 |
| AUSTRIA | NORTH MACEDONIA | 1 |
| AUSTRIA | SLOVAKIA | 1 |
| BRAZIL | AUSTRIA | 1 |
| BRAZIL | BELGIUM | 3 |
| BRAZIL | CUBA | 1 |
| BRAZIL | FRANCE | 1 |
| BRAZIL | ISRAEL | 1 |
| BRAZIL | PORTUGAL | 1 |
| BRAZIL | SPAIN | 1 |
| BRAZIL | SWEDEN | 1 |
| CANADA | AUSTRALIA | 1 |
| CANADA | AUSTRIA | 1 |
| CANADA | BELGIUM | 1 |
| CANADA | BRAZIL | 3 |
| CANADA | DENMARK | 1 |
| CANADA | FRANCE | 2 |
| CANADA | INDIA | 1 |
| CANADA | IRAN | 2 |
| CANADA | IRELAND | 1 |
| CANADA | ISRAEL | 1 |
| CANADA | MALAYSIA | 1 |
| CANADA | NEW ZEALAND | 2 |
| CANADA | SINGAPORE | 1 |
| CANADA | SPAIN | 1 |
| CANADA | SWEDEN | 1 |
| CHILE | ARGENTINA | 1 |
| CHILE | PARAGUAY | 1 |
| CHINA | AUSTRALIA | 9 |
| CHINA | BANGLADESH | 1 |
| CHINA | CANADA | 13 |
| CHINA | CZECH REPUBLIC | 1 |
| CHINA | DENMARK | 1 |
| CHINA | EGYPT | 1 |
| CHINA | GERMANY | 3 |
| CHINA | GREECE | 1 |
| CHINA | INDIA | 3 |
| CHINA | INDONESIA | 2 |
| CHINA | IRAN | 1 |
| CHINA | ISRAEL | 2 |
| CHINA | ITALY | 1 |
| CHINA | JAPAN | 8 |
| CHINA | KOREA | 1 |
| CHINA | POLAND | 1 |
| CHINA | SOUTH AFRICA | 1 |
| CHINA | SPAIN | 1 |
| CHINA | SWEDEN | 5 |
| CHINA | UNITED KINGDOM | 5 |
| CHINA | USA | 84 |
| CROATIA | SLOVENIA | 1 |
| CZECH REPUBLIC | ALBANIA | 1 |
| CZECH REPUBLIC | AUSTRIA | 1 |
| CZECH REPUBLIC | BELGIUM | 1 |
| CZECH REPUBLIC | GREECE | 2 |
| CZECH REPUBLIC | LITHUANIA | 1 |
| CZECH REPUBLIC | MACEDONIA | 1 |
| CZECH REPUBLIC | NORTH MACEDONIA | 1 |
| CZECH REPUBLIC | SLOVAKIA | 1 |
| CZECH REPUBLIC | SWITZERLAND | 1 |
| DENMARK | IRELAND | 1 |
| EGYPT | OMAN | 1 |
| EGYPT | SAUDI ARABIA | 2 |
| FINLAND | AUSTRIA | 1 |
| FINLAND | NORWAY | 1 |
| FRANCE | AUSTRALIA | 1 |
| FRANCE | AUSTRIA | 2 |
| FRANCE | CHILE | 1 |
| FRANCE | FINLAND | 1 |
| FRANCE | HUNGARY | 1 |
| FRANCE | IRELAND | 1 |
| FRANCE | ISRAEL | 2 |
| FRANCE | LEBANON | 1 |
| FRANCE | MEXICO | 1 |
| FRANCE | PARAGUAY | 1 |
| FRANCE | PORTUGAL | 1 |
| FRANCE | SPAIN | 4 |
| FRANCE | SWEDEN | 3 |
| FRANCE | SWITZERLAND | 1 |
| GERMANY | ALBANIA | 1 |
| GERMANY | AUSTRALIA | 1 |
| GERMANY | AUSTRIA | 5 |
| GERMANY | BRAZIL | 1 |
| GERMANY | CAMEROON | 1 |
| GERMANY | CANADA | 4 |
| GERMANY | CHILE | 1 |
| GERMANY | CROATIA | 1 |
| GERMANY | CZECH REPUBLIC | 1 |
| GERMANY | DENMARK | 2 |
| GERMANY | FINLAND | 1 |
| GERMANY | FRANCE | 2 |
| GERMANY | HUNGARY | 2 |
| GERMANY | ISRAEL | 2 |
| GERMANY | LITHUANIA | 1 |
| GERMANY | MACEDONIA | 1 |
| GERMANY | MALAYSIA | 3 |
| GERMANY | NORTH MACEDONIA | 1 |
| GERMANY | POLAND | 3 |
| GERMANY | PORTUGAL | 1 |
| GERMANY | RUSSIA | 2 |
| GERMANY | SERBIA | 1 |
| GERMANY | SINGAPORE | 2 |
| GERMANY | SLOVENIA | 1 |
| GERMANY | SPAIN | 1 |
| GERMANY | SWEDEN | 4 |
| GERMANY | SWITZERLAND | 5 |
| GERMANY | TURKEY | 1 |
| GREECE | BELGIUM | 1 |
| GREECE | CYPRUS | 1 |
| GREECE | PORTUGAL | 1 |
| GREECE | RUSSIA | 1 |
| GREECE | SLOVENIA | 1 |
| HUNGARY | CROATIA | 1 |
| HUNGARY | ISRAEL | 1 |
| HUNGARY | PORTUGAL | 1 |
| HUNGARY | SERBIA | 1 |
| HUNGARY | SLOVENIA | 1 |
| HUNGARY | SWITZERLAND | 1 |
| INDIA | AUSTRALIA | 1 |
| INDIA | ECUADOR | 1 |
| INDIA | SAUDI ARABIA | 1 |
| INDIA | SPAIN | 1 |
| IRAN | DENMARK | 1 |
| ISRAEL | AUSTRIA | 1 |
| ISRAEL | CROATIA | 1 |
| ISRAEL | PORTUGAL | 1 |
| ISRAEL | SERBIA | 1 |
| ISRAEL | SLOVENIA | 1 |
| ISRAEL | SWEDEN | 1 |
| ISRAEL | SWITZERLAND | 1 |
| ITALY | ALBANIA | 1 |
| ITALY | AUSTRALIA | 1 |
| ITALY | AUSTRIA | 2 |
| ITALY | CHILE | 1 |
| ITALY | CROATIA | 1 |
| ITALY | CYPRUS | 1 |
| ITALY | CZECH REPUBLIC | 1 |
| ITALY | ECUADOR | 1 |
| ITALY | EGYPT | 1 |
| ITALY | FINLAND | 1 |
| ITALY | FRANCE | 6 |
| ITALY | GERMANY | 3 |
| ITALY | GREECE | 3 |
| ITALY | HUNGARY | 1 |
| ITALY | INDIA | 1 |
| ITALY | IRAN | 1 |
| ITALY | IRELAND | 1 |
| ITALY | ISRAEL | 3 |
| ITALY | LITHUANIA | 1 |
| ITALY | MACEDONIA | 1 |
| ITALY | NORTH MACEDONIA | 1 |
| ITALY | NORWAY | 1 |
| ITALY | PARAGUAY | 1 |
| ITALY | POLAND | 2 |
| ITALY | PORTUGAL | 3 |
| ITALY | RUSSIA | 1 |
| ITALY | SERBIA | 1 |
| ITALY | SLOVENIA | 2 |
| ITALY | SPAIN | 4 |
| ITALY | SWEDEN | 4 |
| ITALY | SWITZERLAND | 5 |
| ITALY | TURKEY | 1 |
| ITALY | UNITED KINGDOM | 10 |
| JAPAN | AUSTRALIA | 1 |
| JAPAN | CANADA | 4 |
| JAPAN | DENMARK | 1 |
| JAPAN | EGYPT | 3 |
| JAPAN | FINLAND | 3 |
| JAPAN | GERMANY | 4 |
| JAPAN | KOREA | 1 |
| JAPAN | NEW ZEALAND | 1 |
| JAPAN | RUSSIA | 1 |
| JAPAN | SINGAPORE | 1 |
| JAPAN | SPAIN | 1 |
| JAPAN | SWITZERLAND | 1 |
| JAPAN | U ARAB EMIRATES | 1 |
| JAPAN | UNITED KINGDOM | 13 |
| KOREA | AUSTRALIA | 5 |
| KOREA | CANADA | 2 |
| KOREA | IRELAND | 1 |
| KOREA | SINGAPORE | 5 |
| KOREA | UNITED KINGDOM | 1 |
| LITHUANIA | ALBANIA | 1 |
| LITHUANIA | MACEDONIA | 1 |
| LITHUANIA | NORTH MACEDONIA | 1 |
| MACEDONIA | ALBANIA | 1 |
| MACEDONIA | NORTH MACEDONIA | 2 |
| MALAYSIA | INDONESIA | 1 |
| NETHERLANDS | AUSTRALIA | 1 |
| NETHERLANDS | AUSTRIA | 2 |
| NETHERLANDS | BELGIUM | 3 |
| NETHERLANDS | BRAZIL | 5 |
| NETHERLANDS | CANADA | 1 |
| NETHERLANDS | CYPRUS | 1 |
| NETHERLANDS | DENMARK | 2 |
| NETHERLANDS | ECUADOR | 1 |
| NETHERLANDS | FINLAND | 1 |
| NETHERLANDS | FRANCE | 2 |
| NETHERLANDS | GERMANY | 7 |
| NETHERLANDS | GREECE | 1 |
| NETHERLANDS | HUNGARY | 3 |
| NETHERLANDS | ICELAND | 3 |
| NETHERLANDS | INDIA | 1 |
| NETHERLANDS | INDONESIA | 1 |
| NETHERLANDS | IRAN | 2 |
| NETHERLANDS | IRELAND | 1 |
| NETHERLANDS | ISRAEL | 1 |
| NETHERLANDS | ITALY | 6 |
| NETHERLANDS | POLAND | 1 |
| NETHERLANDS | PORTUGAL | 1 |
| NETHERLANDS | RUSSIA | 1 |
| NETHERLANDS | SINGAPORE | 1 |
| NETHERLANDS | SLOVENIA | 1 |
| NETHERLANDS | SPAIN | 1 |
| NETHERLANDS | SWEDEN | 1 |
| NETHERLANDS | SWITZERLAND | 5 |
| NETHERLANDS | UNITED KINGDOM | 18 |
| NIGERIA | UGANDA | 1 |
| NORTH MACEDONIA | ALBANIA | 1 |
| NORWAY | DENMARK | 1 |
| NORWAY | LEBANON | 1 |
| NORWAY | PORTUGAL | 1 |
| POLAND | ALBANIA | 1 |
| POLAND | AUSTRIA | 1 |
| POLAND | CYPRUS | 1 |
| POLAND | CZECH REPUBLIC | 1 |
| POLAND | GREECE | 1 |
| POLAND | LITHUANIA | 1 |
| POLAND | MACEDONIA | 1 |
| POLAND | NORTH MACEDONIA | 1 |
| POLAND | PORTUGAL | 1 |
| POLAND | RUSSIA | 1 |
| POLAND | SLOVENIA | 1 |
| POLAND | SWEDEN | 1 |
| POLAND | SWITZERLAND | 1 |
| POLAND | TURKEY | 1 |
| PORTUGAL | COSTA RICA | 1 |
| PORTUGAL | CROATIA | 1 |
| PORTUGAL | CYPRUS | 1 |
| PORTUGAL | SERBIA | 1 |
| PORTUGAL | SLOVENIA | 2 |
| RUSSIA | CYPRUS | 1 |
| RUSSIA | PORTUGAL | 2 |
| RUSSIA | SLOVENIA | 1 |
| SAUDI ARABIA | OMAN | 1 |
| SERBIA | CROATIA | 1 |
| SERBIA | MACEDONIA | 1 |
| SERBIA | NORTH MACEDONIA | 1 |
| SERBIA | SLOVENIA | 1 |
| SINGAPORE | MALAYSIA | 4 |
| SINGAPORE | NEW ZEALAND | 1 |
| SLOVENIA | CYPRUS | 1 |
| SPAIN | AUSTRIA | 2 |
| SPAIN | CHILE | 1 |
| SPAIN | FINLAND | 1 |
| SPAIN | GREECE | 1 |
| SPAIN | ISRAEL | 1 |
| SPAIN | MEXICO | 1 |
| SPAIN | NEW ZEALAND | 1 |
| SPAIN | PARAGUAY | 1 |
| SPAIN | POLAND | 1 |
| SPAIN | PORTUGAL | 2 |
| SPAIN | RUSSIA | 1 |
| SPAIN | SWEDEN | 2 |
| SPAIN | SWITZERLAND | 1 |
| SWEDEN | ALBANIA | 1 |
| SWEDEN | AUSTRIA | 3 |
| SWEDEN | CZECH REPUBLIC | 2 |
| SWEDEN | DENMARK | 1 |
| SWEDEN | FINLAND | 4 |
| SWEDEN | LITHUANIA | 1 |
| SWEDEN | MACEDONIA | 1 |
| SWEDEN | NORTH MACEDONIA | 1 |
| SWEDEN | SWITZERLAND | 2 |
| SWITZERLAND | ALBANIA | 1 |
| SWITZERLAND | AUSTRIA | 2 |
| SWITZERLAND | CROATIA | 1 |
| SWITZERLAND | DENMARK | 2 |
| SWITZERLAND | FINLAND | 2 |
| SWITZERLAND | GREECE | 2 |
| SWITZERLAND | IRELAND | 1 |
| SWITZERLAND | LITHUANIA | 1 |
| SWITZERLAND | MACEDONIA | 1 |
| SWITZERLAND | NORTH MACEDONIA | 1 |
| SWITZERLAND | PORTUGAL | 1 |
| SWITZERLAND | SERBIA | 1 |
| SWITZERLAND | SLOVENIA | 1 |
| TURKEY | ALBANIA | 1 |
| TURKEY | AUSTRIA | 1 |
| TURKEY | CZECH REPUBLIC | 1 |
| TURKEY | LITHUANIA | 1 |
| TURKEY | MACEDONIA | 1 |
| TURKEY | NORTH MACEDONIA | 1 |
| TURKEY | SAUDI ARABIA | 1 |
| TURKEY | SWEDEN | 1 |
| TURKEY | SWITZERLAND | 1 |
| UNITED KINGDOM | AUSTRALIA | 6 |
| UNITED KINGDOM | AUSTRIA | 2 |
| UNITED KINGDOM | BELGIUM | 2 |
| UNITED KINGDOM | BRAZIL | 2 |
| UNITED KINGDOM | CANADA | 5 |
| UNITED KINGDOM | CHILE | 2 |
| UNITED KINGDOM | CYPRUS | 1 |
| UNITED KINGDOM | DENMARK | 4 |
| UNITED KINGDOM | FINLAND | 3 |
| UNITED KINGDOM | FRANCE | 9 |
| UNITED KINGDOM | GERMANY | 6 |
| UNITED KINGDOM | GREECE | 2 |
| UNITED KINGDOM | HUNGARY | 2 |
| UNITED KINGDOM | IRAN | 2 |
| UNITED KINGDOM | IRELAND | 3 |
| UNITED KINGDOM | ISRAEL | 2 |
| UNITED KINGDOM | NEW ZEALAND | 2 |
| UNITED KINGDOM | NORWAY | 2 |
| UNITED KINGDOM | POLAND | 1 |
| UNITED KINGDOM | PORTUGAL | 1 |
| UNITED KINGDOM | RUSSIA | 1 |
| UNITED KINGDOM | SAUDI ARABIA | 3 |
| UNITED KINGDOM | SINGAPORE | 1 |
| UNITED KINGDOM | SLOVENIA | 1 |
| UNITED KINGDOM | SPAIN | 4 |
| UNITED KINGDOM | SWEDEN | 8 |
| UNITED KINGDOM | SWITZERLAND | 7 |
| USA | ALBANIA | 1 |
| USA | AUSTRALIA | 5 |
| USA | AUSTRIA | 5 |
| USA | BANGLADESH | 1 |
| USA | BELGIUM | 1 |
| USA | BRAZIL | 6 |
| USA | CANADA | 15 |
| USA | CROATIA | 1 |
| USA | CYPRUS | 1 |
| USA | CZECH REPUBLIC | 3 |
| USA | DENMARK | 5 |
| USA | ECUADOR | 3 |
| USA | EGYPT | 2 |
| USA | FINLAND | 1 |
| USA | FRANCE | 3 |
| USA | GERMANY | 18 |
| USA | GREECE | 5 |
| USA | HUNGARY | 9 |
| USA | ICELAND | 3 |
| USA | INDIA | 6 |
| USA | IRAN | 3 |
| USA | IRELAND | 4 |
| USA | ISRAEL | 10 |
| USA | ITALY | 23 |
| USA | JAPAN | 24 |
| USA | KOREA | 18 |
| USA | LEBANON | 1 |
| USA | LITHUANIA | 1 |
| USA | MACEDONIA | 1 |
| USA | NETHERLANDS | 25 |
| USA | NEW ZEALAND | 3 |
| USA | NORTH MACEDONIA | 1 |
| USA | NORWAY | 2 |
| USA | POLAND | 2 |
| USA | PORTUGAL | 3 |
| USA | RUSSIA | 1 |
| USA | SAUDI ARABIA | 3 |
| USA | SERBIA | 1 |
| USA | SINGAPORE | 3 |
| USA | SLOVAKIA | 1 |
| USA | SLOVENIA | 2 |
| USA | SPAIN | 9 |
| USA | SWEDEN | 7 |
| USA | SWITZERLAND | 5 |
| USA | TURKEY | 3 |
| USA | U ARAB EMIRATES | 2 |
| USA | UNITED KINGDOM | 22 |

**Method**

**Citespace**

CiteSpace 6.4.R1 was employed to conduct knowledge-mapping analyses, including keyword co-occurrence, reference co-citation, author co-citation, and burst detection. The data source was set to Web of Science (WoS). The time span for analysis was defined from January 2015 to December 2025, with the time slicing parameter set to 1 year to ensure high temporal resolution.

In the Text Processing panel, Title, Abstract, Author Keywords (DE), and Keywords Plus (ID) were selected as the term sources. Noun Phrases was chosen as the term type to extract representative topic terms, and Burst Terms detection was enabled to identify terms with strong temporal citation bursts.

For Node Types, Keyword, Reference, Cited Author, and Cited Journal were selected to construct keyword co-occurrence networks, reference co-citation networks, author co-citation networks, and journal co-citation networks. The link strength was set to Cosine, and the network scope was set to Within Slices, meaning that networks were generated for each time slice and subsequently merged.

In the Selection Criteria settings, the g-index was applied as the thresholding method, with the scale factor k set to 25, which helps maintain an appropriate balance between network representativeness and structural clarity. Burst detection employed CiteSpace’s default implementation of the Kleinberg algorithm.

Pruning procedures included the Pathfinder and Minimum Spanning Tree (MST) algorithms, as well as pruning of the merged network, in order to remove redundant links and enhance the structural visibility of clusters. These pruning techniques improve interpretability by highlighting essential connections while minimizing noise.

All visualizations, including cluster views, timeline views, and burst detection maps, were generated automatically by CiteSpace based on the configured parameters, providing a structured representation of the field’s knowledge base and emerging research fronts.

**VOSviewer**

VOSviewer (version 1.6.19) was used to perform keyword co-occurrence analysis and generate visual knowledge maps. In the “Create Map” module, the type of analysis was set to Co-occurrence, and the unit of analysis was defined as All keywords, which includes both author keywords and index keywords. The counting method was set to Full counting, meaning that each keyword occurrence was counted equally regardless of the number of co-occurring keywords within a single document.

To ensure robustness and reduce noise in the network, a minimum occurrence threshold was applied. The threshold value was set to 70 occurrences, and of the 21,261 extracted keywords, 357 met this criterion and were included in the final co-occurrence network. VOSviewer automatically calculated the co-occurrence strength between keyword pairs and generated a network visualization based on the association strength normalization method. In the visual maps, node size represents the frequency of keyword occurrence, link thickness indicates the co-occurrence strength, and different colors correspond to clusters identified by the software. These procedures allowed for the identification of major research themes, cluster structures, and the conceptual organization of the field.
